# Mechanism of superior luminescent and high-efficiency photocatalytic properties of Eu-doped calcium aluminate by low-cost self-propagating combustion synthesis technique

**DOI:** 10.1038/s41598-017-03099-9

**Published:** 2017-06-06

**Authors:** Jiancun Rao, Yujin Wang, Wen Wang, Hua Ke, Yechen Li, Yang Zhao, Zhiliang Diao, Dechang Jia, Yu Zhou

**Affiliations:** 1 Institute for Advanced Ceramics, School of Materials Science and Engineering, Harbin Institute of Technology, Harbin, 150001 P.R. China; 2 AIM Lab, Maryland NanoCenter, University of Maryland, College Park, MD-20742 USA

## Abstract

Eu-doped calcium aluminate was synthesized via the low-cost self-propagating combustion synthesis (SPCS) technique, whose phase constitutions were identified as Ca_9_Al_6_O_18_ and Ca_3_Al_2_O_6_. The Ca^2+^ ions in Ca_9_Al_6_O_18_ rather than Ca_3_Al_2_O_6_ phase were replaced by Eu^3+^ ions. The product exhibits the superior luminescent property and photocatalytic activity, which may find potential applications in the display devices and environment treatments.

## Introduction

In recent years, the energy conversion and environmental issues have attracted more extensive attention. Therefore, exploiting the green functional materials and utilizing non-polluting renewable energy are gradually becoming main topics. The functional materials doped with rare earth ions have been widely used in aviation, construction, electronics, optical conversion device and bio-molecular probe fields because of their advantages of low-cost, pollution-free, and nontoxic^1–5^, for instance, the rare earth permanent magnetic materials^6^, the rare earth superconducting materials^7^, the rare earth hydrogen storage material^8^ and so on.

Aluminates are a kind of low-cost, stable, anti-radiation and eco-friendly host of the rare earth luminescence materials^9–15^. However, one hand, the synthesis temperature of aluminates is usually very high and reaches up to 1200–1600 °C when using the traditional high-temperature calcination method by oxides (*e.g.* CaO and Al_2_O_3_) as starting material in the industry. On the other hand, the obtained product is also usually composed of mixed phases of multiple aluminates such as CaAl_2_O_4_, CaAl_4_O_7_, CaAl_12_O_19_, Ca_3_Al_2_O_6_, Ca_12_Al_14_O_13_ and so on, the mixed phase identification of which is hardly been discussed in the past researches. Recently, the SPCS technique was widely applied to synthesize inorganic materials, which can effectively reduce the reaction temperature and shorten the reaction time. For example, Rafiaei *et al*. prepared Gd and Eu ions doped Y_2_O_3_ materials, respectively, which exhibited superior luminescent emission property^16, 17^.

In this study, a functional material of Eu-doped calcium aluminate was synthesized by the SPCS technique, which realized the obviously fast reaction at low temperature with low energy consumption compared with the traditional high-temperature calcination method. The product presents not only the superior luminescent property but also high-efficiency photocatalytic activity, which casts a potential application in the display devices and environment treatments. The phase constitution is studied by X-ray Diffraction (XRD) pattern, Fourier Translation Infrared (FT-IR) spectrum and transmission electron microscopy (TEM) *etc*. approaches, as well as the luminescent and photocatalytic mechanisms are also discussed in detail.

## Results and Discussion

**Figure 1:**
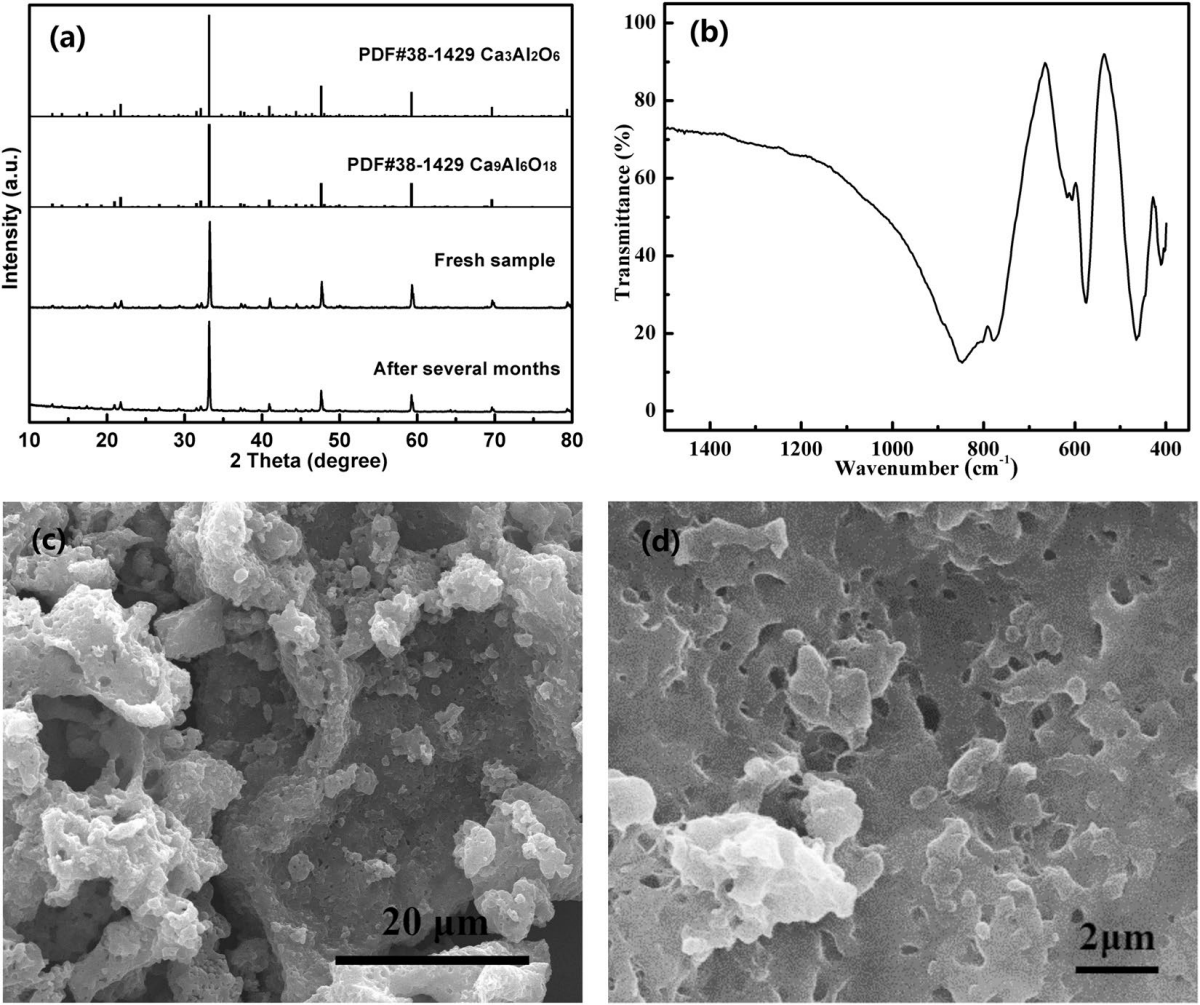
XRD pattern (**a**), FT-IR spectrum (**b**), SEM images (**c**,**d**) of the as-prepared Eu-doped calcium aluminate.

Figure 1(a) shows the XRD pattern of the as-prepared Eu-doped calcium aluminate, which agrees well with the standard spectrum of both Ca_9_Al_6_O_18_ (PDF #70-0839) and Ca_3_Al_2_O_6_ (PDF #38-1429). No other miscellaneous diffraction peaks are observed, indicating that the as-prepared sample is completely converted to Ca_3_Al_2_O_6_ or/and Ca_9_Al_6_O_18_ crystalline phases without generating other types of calcium aluminates. In addition, these strong, narrow and sharp diffraction peaks reveal that the sample has a high degree of crystallinity. It is carefully analyzed that the angles of two strong peaks at 2*θ* = 33.26° and 2*θ* = 47.72°are slightly increased compared with the standard spectrum, which demonstrates that the doped crystal lattices are slightly contracted owing to the effect of some Eu^3+^ replaced Ca^2+^ ions, considering the ionic diameter of Ca^2+^ and Eu^3+^ are 0.099 and 0.095 nm, respectively. Besides, the metallic element contents in the sample were detected by inductive coupling plasma emission spectrograph (ICP-AES). The results show that the atom ratio of Al, Ca and Eu was 3.9: 6.6: 0.02, which was close to theoretical content in the sample.

The FT-IR spectrum of Eu-doped calcium aluminate sample is also performed. It was reported that the absorption band of AlO_4_ and AlO_6_ in condensed matter locates in the range of 900–700 cm^−1^ and 680–500 cm^−1^, respectively^18^. As we can see from the spectrum, the strong absorption band at 900–600 cm^−1^ in Fig. 1(b) can be attributed to the AlO_4_ vibration, which coincides with the corner-sharing tetrahedron AlO_4_ structure in the Ca_3_Al_2_O_6_ or/and Ca_9_Al_6_O_18_ crystals. Two groups of absorption at 600–400 cm^−1^ are derived from characteristic vibration of Al-O bond^19^. It proves that the lattice structures of Ca_3_Al_2_O_6_ or/and Ca_9_Al_6_O_18_ crystals have not changed but with slight distortion, and the Ca^2+^ ions rather than Al^3+^ are replaced by Eu^3+^. In addition, the absorption bands at 1500 cm^−1^ and 3450 cm^−1^ occurs due to the vibration from CO_3_^2−^ and OH^−^ groups, respectively, because of CO_2_ and H_2_O in the air. The morphology observed in SEM, as shown in Fig. 1(c), indicates significant aggregate and the particle shape is generally irregular due to the high-temperature calcination. Fig. 1(d) confirmed the porous feature. This may be caused by the urea combustion which leading to the gases releasing and then inhibiting the formation of dense bulk samples in the SPCS process.

**Figure 2:**
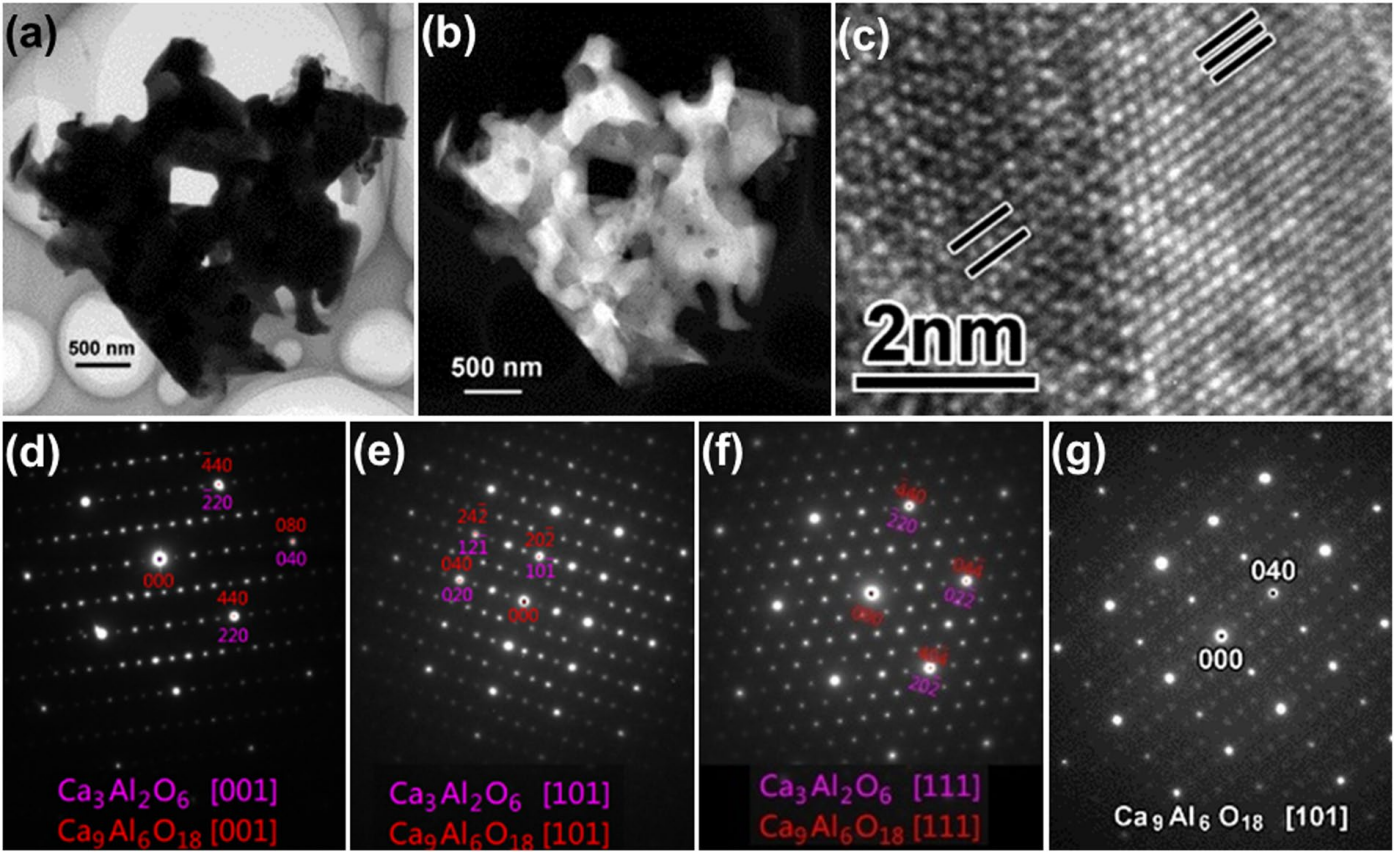
TEM analysis results of the as-prepared Eu-doped calcium aluminate sample. (**a**) BF image, (**b**) HAADF image, (**c**) HRTEM image, (**d**,**e**,**f**,**g**) EDPs from 3 different axes. The HRTEM image is corresponding to [111] EDP.

Figure 2 gives the TEM analysis results of the as-prepared Eu-doped calcium aluminate sample. Fig. 2(a) and (b) are bright field (BF) and high-angle annular dark field (HAADF) images, respectively, from the same aggregation. It is obvious that there are two phases with different contrast, which intersperse with each other. The selected area electron diffraction patterns (EDPs) obtained by careful tilting are shown in Fig. 2(d,e and f). These EDPs can be assigned as either Ca_3_Al_2_O_6_ or Ca_9_Al_6_O_18_ phase. It is very difficult to distinguish the isolated Ca_3_Al_2_O_6_ or Ca_9_Al_6_O_18_ phase due to their interspersion. The strongest spots in most EDPs can be assigned as both the two phases, only with the differences of the indices, as shown in the image of Fig. 2(d,e and f). Further TEM study reveals part of the aggregated particles are pure Ca_9_Al_6_O_18_ phase. This can be proved by the EDP, as shown in Fig. 2(g), which is from the adjacent area. The intensities of the strongest spots in Fig. 2(g), *e.g.* (000) and (040), are approximately the same. While in Fig. 2(e) the intensities of those spots, like (000) and (040), is not the same, and the stronger spots, like (020), are also from both the two phases as the strongest spots do. But the weaker spots are only from Ca_9_Al_6_O_18_ phase. The HRTEM image in Fig. 2(c), which is corresponding to the EDP in Fig. 2(f), shows the interface between Ca_3_Al_2_O_6_ (right side) and Ca_9_Al_6_O_18_ (left side) phases. The {101} planes of both the two phases are labeled by the black solid lines in the image. The increment of the lattice parameters of Ca_9_Al_6_O_18_ phase is clearly illustrated by these lines.

**Figure 3:**
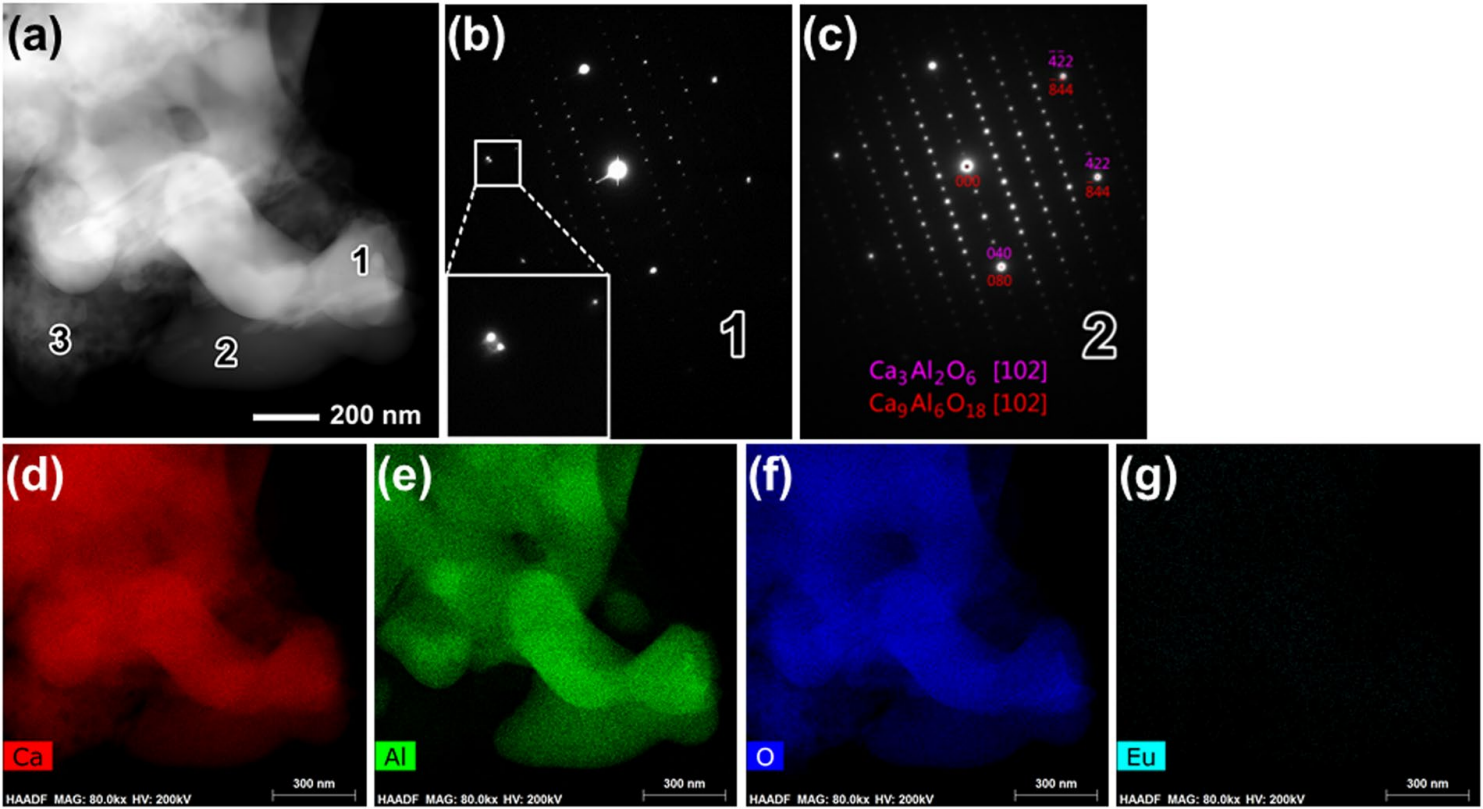
Detailed TEM analysis results of the as-prepared Eu-doped calcium aluminate sample. (**a**) HAADF image, (**b**) EDP from spot marked by “1” in (**a**), (**c**) EDP from spot “2”, (**d**–**g**) elementary maps of Ca, Al, O and Eu, respectively.

The brighter part in the BF image, while darker in HAADF image, is proved as mostly Ca_3_Al_2_O_6_ phase by EDP. Meanwhile, the other contrast is mostly Ca_9_Al_6_O_18_ phase. Normally these two phases are formed exactly in the same crystallographic orientation. So the EDPs can be assigned as both as Ca_3_Al_2_O_6_ and Ca_9_Al_6_O_18_ phases. However, the fewer cases occur that these two phases are almost, but not completely, in the same crystallographic direction. Figure 3 shows a detailed TEM analysis result for this case. Figure 3(a) is the HAADF image from one corner of the aggregated particles. The EDPs from area marked with numbers “1” and “2”, darker and brighter areas as discussed above, are shown in Fig. 3(b and c), respectively. One enlarged insert in Fig. 3(b) shows clearly two discrete spots from these two phases separately. It is obvious that Ca_3_Al_2_O_6_ and Ca_9_Al_6_O_18_ phases are not perfectly in the same crystallographic direction in area “1”. Accordingly, Ca_3_Al_2_O_6_ and Ca_9_Al_6_O_18_ phases in area “2” are exactly in the same direction as EDP shown in Fig. 3(c). The elementary maps of Ca, Al, O and Eu are shown in Fig. 3(d–g), respectively. From these elementary maps, it is obvious that the distribution of Ca, Al and O, even the rare Eu, are very homogenous. There seems to be no differences between Ca_3_Al_2_O_6_ and Ca_9_Al_6_O_18_ phases. So quantitative EDS analysis should be done carefully and profoundly.

Table 1 gives the chemical constitution (at. %) by the energy dispersive X-ray spectroscopy (EDS) analysis from those three different spots marked by numbers “1”, “2” and “3” in Fig. 3(a). It was confirmed that the relative content of Eu is higher in Ca_9_Al_6_O_18_ than in Ca_3_Al_2_O_6_ phase. The content of Eu is slightly higher in spot “2” than in “1” but with a lower Ca percentage. This verifies that the Eu^3+^ replaced Ca^2+^ in the particles. Due to the randomness of Eu^3+^ replacing Ca^2+^, it is necessary to have a large unit cell to contain these Eu^3+^ ions. Then there was formed as the Ca_9_Al_6_O_18_ phase, which the lattice parameter is doubled as that of Ca_3_Al_2_O_6_ phase. The replacement of Eu^3+^ for Ca^2+^ results in a different space group of Ca_9_Al_6_O_18_ phase, which can be proved by the occurrence of structural-extinction-spots, like (010) spot, in Figs 2(a) and 3(c). Based on this point, the structure of Ca_9_Al_6_O_18_ phase is preferred by the authors.

**Table 1:** Chemical constitution (at. %) of the particles in Fig. 3(a) by EDS analysis.

spot	Ca	Al	O	Eu
1	12.760	28.701	58.439	0.099
2	9.949	27.606	62.788	0.114
3	81.335	4.782	13.882	0.000

**Figure 4:**
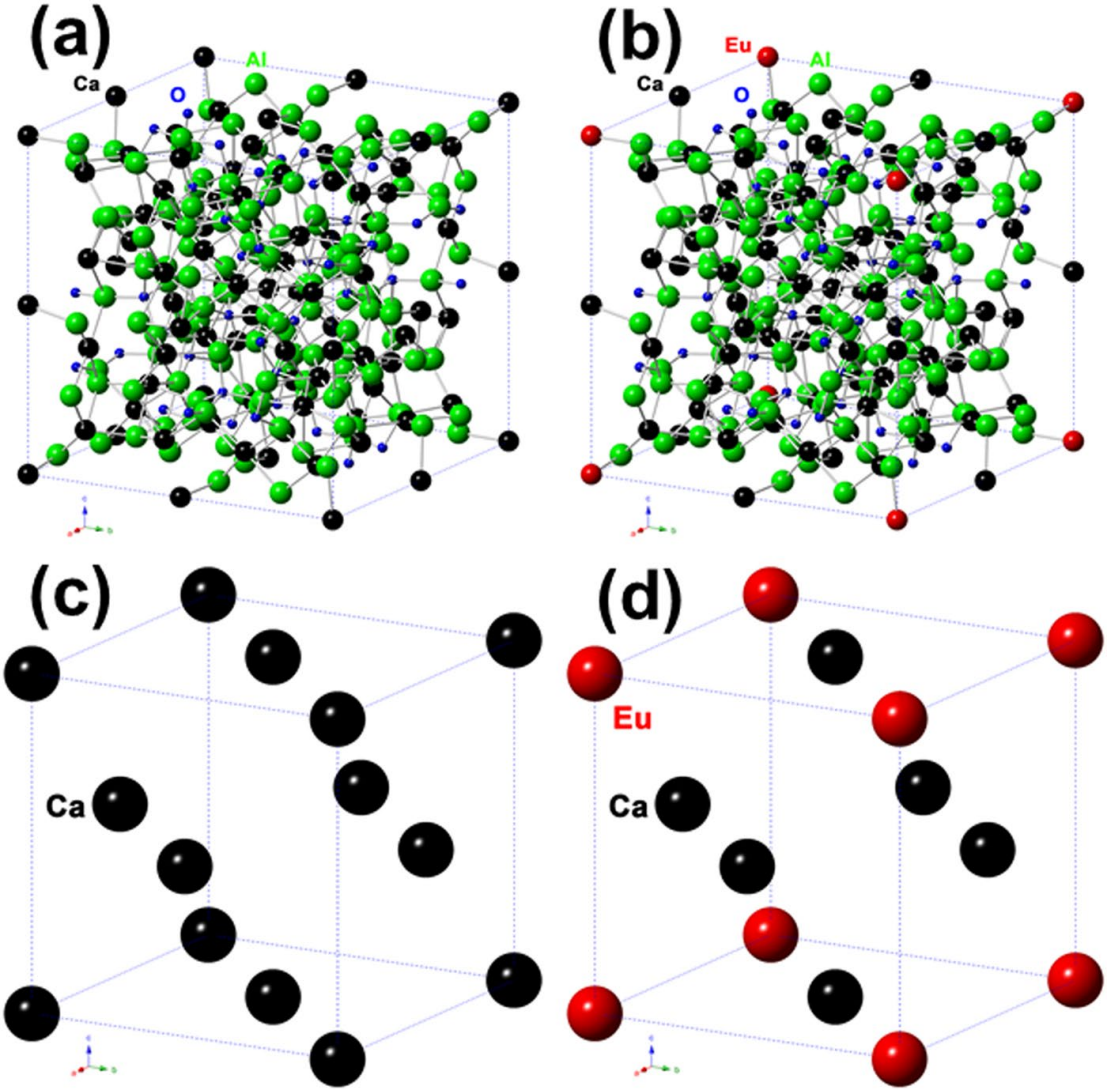
The schematic diagram of parts of Ca replaced by Eu in the unit cell of Ca_9_A_l6_O_18_ phase. (**a**) Original unit cell structure of Ca_9_Al_6_O_18_, (**b**) parts of Ca replaced by Eu, (**c**) only Ca at 4*a* positions for clarity, (**d**) Ca at 8 corners replaced by Eu.

We know that the unit cell of Ca_9_Al_6_O_18_ contains 72 Ca, 48 A1 and 144 O atoms. The structure is built of six-fold rings centered on three-fold axes and composed of two types of distorted AlO_4_ tetrahedra. The holes in between the rings contain the Ca atoms. In the unit cell there are 80 such possible holes; 72 of them are filled up with Ca atoms leaving 8 vacant on threefold axes at position of (1/8, 1/8, 1/8) together with its symmetry-related positions. The extra atoms could fit into these 8 holes^20^. Since the sizes of Ca^2+^ and Eu^3+^ are comparable (0.099 nm and 0.095 nm, respectively), it is almost certain that there are two possible ways for Eu^3+^ ions to stay in the unit structure. First, Eu^3+^ ions could replace some of the Ca^2+^ ions and leaving some Ca^2+^ vacancies for balancing charges. Second, Eu^3+^ ions could go into these 8 holes of the structure. If Eu^3+^ ion occupies vacancies site such as (1/8, 1/8, 1/8), there should be some new vacancies of Ca^2+^ as near as possible in order to achieve a localized balancing of charges. The latter would probably not happen for the symmetry of the unit cell structure. So only small amount of Ca^2+^ ions were replaced by Eu^3+^ ions, which is already proved by FT-IR spectrum in Fig. 1(b). A schematic diagram is given in Fig. 4 showing the unit cell of Ca_9_A_l6_O_18_ phase and the replacement of parts of Ca by Eu. Figure 4(a) is the original unit cell of Ca_9_A_l6_O_18_ phase and Fig. 4(b) is the final result of the unit cell with parts of Ca replaced by Eu. Figure. 4(c,d) show only Ca atoms at 4*a* positions (Wyckoff position) for clarity as well as its partial replacement by Eu in the unit cell. This also matches the results of very little Eu doped into Ca_9_A_l6_O_18_ phase.

There are six types of Ca atoms in the structure of Ca_9_Al_6_O_18_. Two of them form distorted octahedra with O. The distortion in the octahedral arrangement of oxygen atoms and the presence of short Ca-O bonds indicates a certain amount of strain and hence there will be potential energy stored up in the structure. This potential energy might be available to assist the break-up of the structure by the action like water. So the “flash set” phenomenon of Ca_3_Al_2_O_6_ is attributed to its high reactivity with water^20^. While in our products, the Eu doped calcium aluminate (Ca_3_Al_2_O_6_ and Ca_9_Al_6_O_18_) is quite stable. The XRD pattern of the powder leaving in the room for months is almost the same as that from just-produced powder. This is also one advantage of our products. It should be noted that there are fragments on the aggregated particles, *e.g.* Area “3” in Fig. 3(a). The EDS result from this kind of fragment shows that it may be only calcium oxide (CaO) phase with very little Al solidified inside. There is no Eu detected by EDS in these fragments. This implies that it is not so easy for Eu^3+^ to substitute for Ca^2+^ in calcium oxide due to the strong bond between Ca^2+^ and O^2−^. While it becomes easier in more complicated structure of Ca_9_Al_6_O_18_ phase as discussed above.

**Figure 5:**
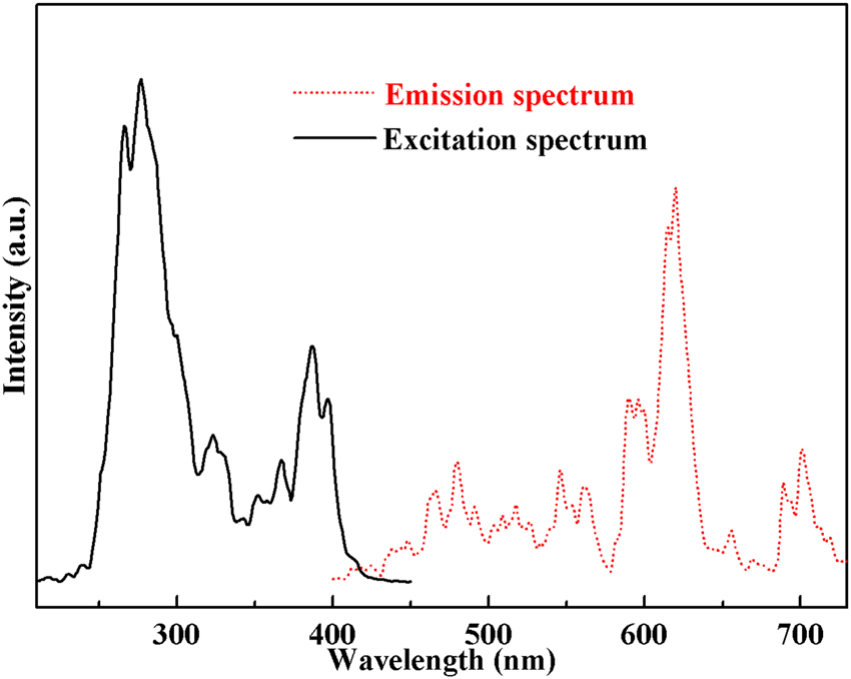
Excitation (*λ*_em_ = 620 nm) and emission (*λ*_ex_ = 277 nm) spectra of Eu-doped calcium aluminate sample.

The luminescent property of the as-prepared Eu-doped calcium aluminate sample is investigated. As the emission spectrum shown in Fig. 5 (*λ*_ex_ = 277 nm), the sample emits characteristic red light. The emissions at (590 nm, 596 nm), (615 nm, 620 nm), (656 nm, 669 nm), and (689 nm, 701 nm) stem from ^5^D_0_ → ^7^F_1_, ^5^D_0_ → ^7^F_2_, ^5^D_0_ → ^7^F_3_ and ^5^D_0_ → ^7^F_4_ transitions (4f → 4f), respectively. Generally, the magnetic dipole transition ^5^D_0_ → ^7^F_1_ is permitted. The electric dipole transition ^5^D_0_ → ^7^F_2_ is so sensitive to symmetry that it exceptionally allowed if Eu^3+^ ion occupies a non-inversion center site. Therefore, the strongest transition ^5^D_0_ → ^7^F_2_ at 620 nm indicates that Eu^3+^ ions locate non-inversion center sites in the calcium aluminate host. Moreover, Eu^3+^ ions are very sensitive to the crystal field. Owing to 4f electronic configuration interfused opposite 5d and crystal field asymmetry, f → f forbidden transition can be partly relaxed, which results in more intensive ^5^D_0_ → ^7^F_2_ transition than ^5^D_0_ → ^7^F_1_^21^. In addition, Multi-state ^7^F_J_ of Eu^3+^ is split into multiple stark energy levels under the crystal field effect, so considerable splitting results appear in four groups of emission peaks of the ^5^D_0_ → ^7^F_J_. The excitation spectrum of the sample by monitoring ^5^D_0_ → ^7^F_2_ transition is also carried out (Fig. 5). The peaks at 320–400 nm ascribe to the intra-configurational 4f → 4f transition of Eu^3+^ ions, in which the strong absorption at 387 nm belongs to ^7^F_0_ → ^5^L_6_ transition. Additionally, the strong absorption at 277 nm drives from the calcium aluminate host.

**Figure 6:**
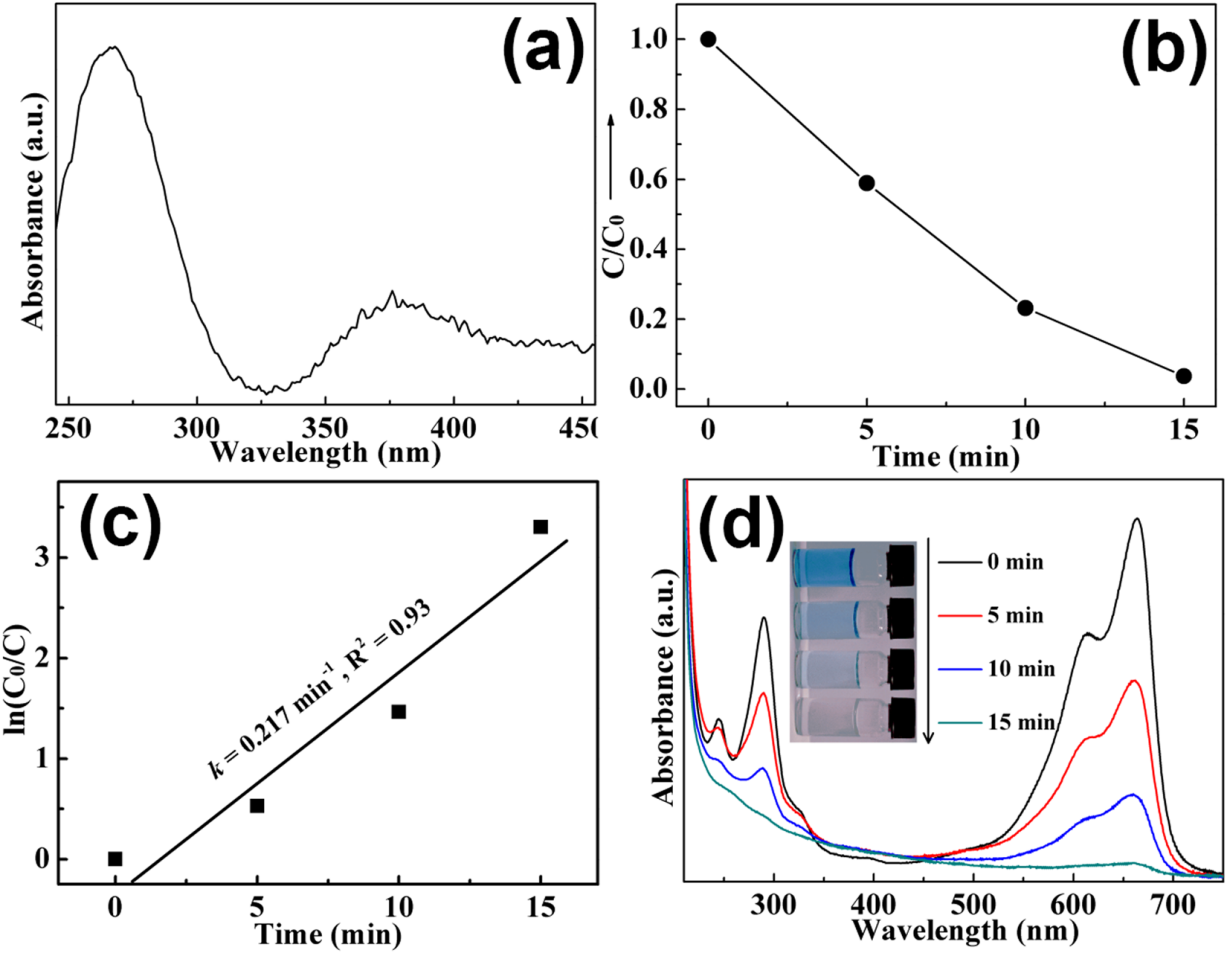
UV-vis spectrum (**a**) of Eu-doped calcium aluminate sample, dynamic curve (**b**), plots of ln (c_0_/c) versus time (**c**) and absorbance variations (**d**) of MB solution over Eu-doped calcium aluminate sample.

In order to investigate the photocatalytic activity of the as-prepared Eu-doped calcium aluminate sample, the UV-vis DRS of the sample is firstly performed to explore the light absorption property. As shown in Fig. 6(a), the as-prepared sample exhibits two absorption bands, which are in accord with the excitation spectrum in Fig. 5. Therefore, two absorption bands at 200–320 nm and 320–450 nm stem from transition from valence band (VB) to conduction band (CB) of the calcium aluminate host and 4f → 4f transition of Eu^3+^ ions, respectively. The methylene blue (MB) dye is used for target molecules to evaluate the degradation ability of the as-prepared sample. Figure 6(b) shows the dynamic curve of MB degradation. After running 15 min, the degradation rate of MB is more than 98%. Moreover, the kinetic curve of MB degradation can be approximated as a pseudo-first-order process^22–25^.

**Figure 7:**
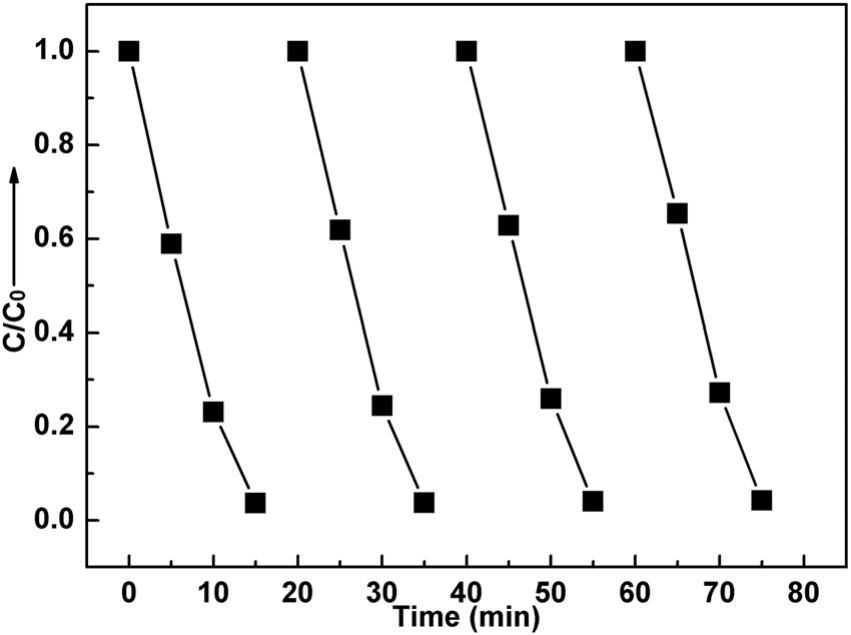
Cycle photodegradation runs of MB solution over Eu-doped calcium aluminate sample.

By plotting the ln(*c*_0_/*c*) versus time and making linear fitting for kinetic curves (Fig. 6(c)), the removal rate constant *k* of MB is estimated to be 0.217 min^−1^. Furthermore, from the absorbance variations of MB solution in the photocatalytic reaction process (Fig. 6(d)), it has no shifting of the maximum absorption wavelength position of MB solution at 663 nm, and the absorption peak at 292 nm in ultraviolet region vanishes besides visible region. It implies that the benzene/heterocyclic rings of MB molecule may be completely decomposed, leading to the thorough mineralization of MB^22–25^. In order to assess the reusability of the sample which is crucial for its practical application, the circle runs experiments of MB solution photodegradation over Eu-doped calcium aluminate sample were performed. As shown in Fig. 7, the results indicated the photocatalytic ability of sample had not obviously loss after four recycles, indicating that the sample exhibits superior stability and durability.

**Figure 8:**
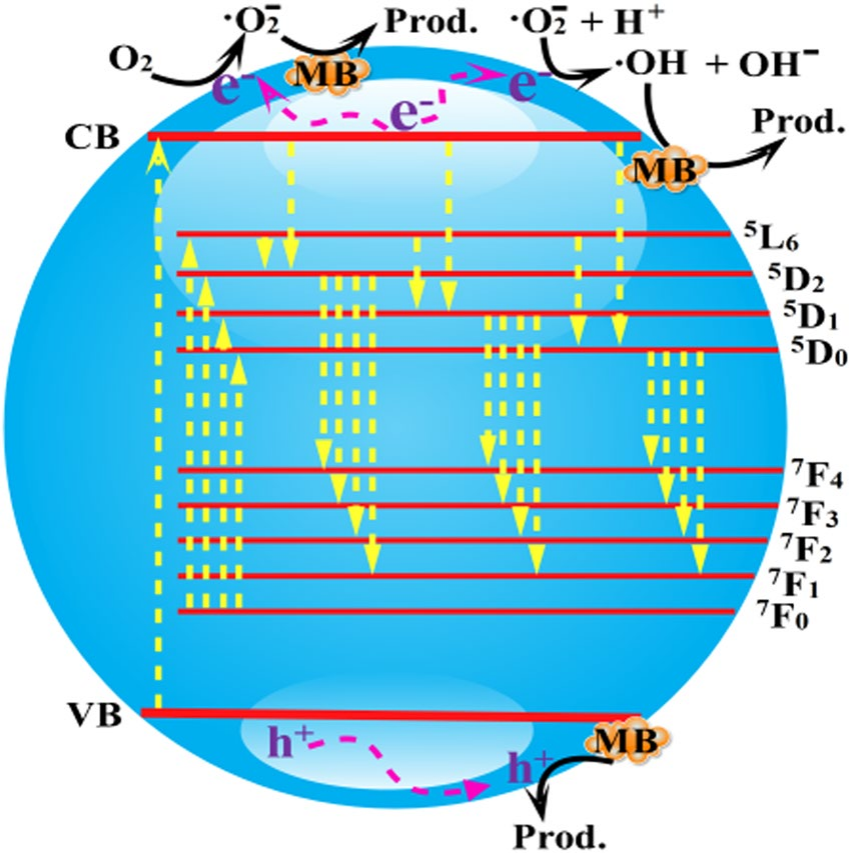
The transfer behavior of charge carriers as well as luminescent and photocatalytic mechanism.

The possible transfer behavior of charge carriers as well as luminescent and photocatalytic mechanism are shown in Fig. 8. Under the light excitation, the calcium aluminate host and Eu^3+^ ions are all excited at the same time. Electrons in the CB of calcium aluminate host and the ground state ^7^F_0_ of Eu^3+^ ions transfer into the corresponding CB and ^5^D_0_, ^5^D_1_, ^5^D_2_ and ^5^L_6_ states of them, respectively. At the luminescent process, electrons in the excitation state ^5^D_1_ and ^5^D_2_ return to ^7^F_1_, ^7^F_2_, ^7^F_3_, ^7^F_4_ states of Eu^3+^ ions to generate luminescence. It should be pointed out that part of the electron in the CB of the calcium aluminate host and ^5^L_6_ states can transfer into ^5^D_0_, ^5^D_1_, ^5^D_2_ states by means of multi-phonon assisted relaxation effect to enhance luminescent property. At the MB degradation process, part of the electrons in the CB of the calcium aluminate host migrate to the sample surface and are captured by O_2_ molecules in water to yield superoxide radicals (•O_2_^−^). The superoxide radicals may further transform into hydroxyl radicals (•OH). Finally, the superoxide radicals, hydroxyl radicals and holes oxidize decompose MB dye.

## Conclusion

A functional material of Eu-doped calcium aluminate was firstly obtained by the self-propagating combustion synthesis technique with low cost and low energy comsuption, which presents a significant decrease in the synthesis temperature compared with the solid-state calcination method using oxides (*e.g.* CaO and Al_2_O_3_) as starting material. The product is composed of two phases, *i.e.* Ca_3_Al_2_O_6_ and Ca_9_Al_6_O_18_, as well as exhibits the superior luminescent property and high-efficiency photocatalytic activity, which may have the potential application prospect for serving as phosphor in the display device and photocatalyst in the environment treatment, respectively.

## Methods

According to the stoichiometric ratio of Ca_2.98_Al_2_O_6_: 0.02Eu^3+^, Eu_2_O_3_ was transferred to 250 mL beaker and dissolved *via* concentrated HNO_3_, then adding Al(NO_3_)_3_•9H_2_O, Ca(NO_3_)_2_•4H_2_O, CO(NH_2_)_2_ and appropriate distilled water. We keep on stirring, dissolving and heating until the solution was evaporated to be viscous. Subsequently, the beaker was put into a muffle furnace and kept at 500 °C. After a few minutes, the mixture started burning and last for 5–7 min. Finally, the obtained precursor was transferred into the corundum crucible and calcined at 900 °C for an additional 6 h to obtain the final white products.

X-ray diffraction (XRD) pattern was recorded by Rigaku D/max-2200 powder diffractometer. FT-IR spectrum was measured on FT-IR360 infrared spectrometer. The morphology was characterized utilizing scanning electron microscope (SEM, FEI Quanta 200FEG) and transmission electron microscope (TEM, FEI Tecnai G^2^ F30) with high angle annular dark field (HAADF) detector. Luminescent spectrum was measured *via* F4500 fluorescence spectrophotometer. The UV-visible diffuse reflectance spectrum (UV-vis DRS) was recorded on UV-vis spectrophotometer (PG, TU-1901).

The methylene blue (MB) solution (10 mg/L, 100 ml) containing 0.1 g sample was irradiated with a 300 W Xe arc lamp. Before the irradiation, it was stirred for 30 min in the dark environment to achieve the adsorption-desorption equilibrium between MB and sample. The absorbance of MB solution was monitored by UV-vis spectrophotometer (PG, TU-1901) every 5 min.
